# Effect of nonpharmacological interventions on poststroke depression: a network meta-analysis

**DOI:** 10.3389/fneur.2024.1376336

**Published:** 2024-04-05

**Authors:** Ying Li, Yuanyuan Wang, Lei Gao, Xiaohan Meng, Qidan Deng

**Affiliations:** ^1^College of Sports Science, Jishou University, Jishou, China; ^2^Cardiac Care Unit, Sir Run Run Hospital, Nanjing Medical University, Nanjing, China; ^3^School of Nursing, Dalian University, Dalian, China; ^4^Department of Intensive Care Unit, Affiliated Qingyuan Hospital, Guangzhou Medical University, Qingyuan People’s Hospital, Qingyuan, China

**Keywords:** stroke, PSD, depression, network meta-analysis, systematic reviews

## Abstract

**Purpose:**

To investigate the effects of nonpharmacological interventions (NPIs) on poststroke depression (PSD) in stroke patients.

**Methods:**

Computer searches were conducted on the PubMed, Embase, Cochrane Library, Web of Science, China National Knowledge Infrastructure (CNKI), China Science and Technology Journal Database (VIP), and Wanfang databases from their establishment to December 2023. The selection was made using the inclusion and exclusion criteria, and 40 articles were included to compare the effects of the 17 NPIs on patients with PSD.

**Results:**

Forty studies involving seventeen interventions were included. The network findings indicated that compared with conventional therapy (COT), superior PSD improvement was observed for cognitive behavioral therapy (CBT) + acupoint acupuncture (CBTA) (mean difference [MD], −4.25; 95% CI, −5.85 to −2.65), team positive psychotherapy (MD, −4.05; 95% CI, −5.53 to −2.58), music therapy (MT) + positive psychological intervention (MD, −2.25; 95% CI, −3.65 to −0.85), CBT (MD, −1.52; 95% CI, −2.05 to −0.99), mindfulness-based stress reduction (MD, −1.14; 95% CI, −2.14 to −0.14), MT (MD, −0.95; 95% CI, −1.39 to −0.52), acupoint acupuncture + MT (AAMT) (MD, −0.69; 95% CI, −1.25 to −0.14). Furthermore, CBT (MD, −3.87; 95% CI, −4.57 to −3.17), AAMT (MD, −1.02; 95% CI, −1.41 to −0.62), acupressure + MT (MD, −0.91; 95% CI, −1.27 to −0.54), and narrative care + acupressure (MD, −0.74; 95% CI, −1.19 to −0.29) demonstrated superior Pittsburgh Sleep Quality Index (PSQI) improvement compared with COT.

**Conclusion:**

Evidence from systematic reviews and meta-analyses suggests that CBTA improves depression in patients with PSD. Moreover, CBT improves sleep in these patients. Additional randomized controlled trials are required to further investigate the efficacy and mechanisms of these interventions.

## Introduction

According to the World Health Organization (WHO) statistics in 2019, stroke is the second leading cause of death, accounting for approximately 11% of total deaths (1). Stroke has a serious impact on multiple functional domains and often leads to disability, affecting patients’ quality of life and leading to negative emotional states (2). Stroke survivors may face significant health challenges and are more likely to experience psychological disorders owing to their severe symptoms and physical disabilities. Depression is one of the most common complications of stroke, with a prevalence rate of 30–33% (3–6). The core symptom cluster includes feelings of low mood, emotional detachment, fatigue, insomnia, feelings of worthlessness, and even suicidal ideation (7–10). Poststroke depression (PSD) negatively affects physical, cognitive, and functional recovery; increases the risk of recurrent vascular events; reduces quality of life; decreases social participation; and increases mortality rates (11, 12). Therefore, it is of utmost importance to identify safe and effective treatment approaches for PSD (13). However, the effectiveness of pharmacological interventions in PSD remains unclear. Furthermore, the use of medications may be further limited by adverse effects, long reaction times, potential drug-related events, and low compliance (14–16). For these reasons, alternative or complementary choices for medication selection are critical to ensure effective management of PSD (17). Nonpharmacological interventions (NPIs) are scientifically based, noninvasive measures for human health that may offer an alternative approach to improving depressive symptoms (17).

A substantial body of evidence supports the effectiveness of NPIs for depression in various clinical populations (15, 18). Several reviews have identified and qualitatively provided evidence for the use of NPIs in PSD (14, 18, 19). In particular, a review suggested that therapeutic approaches such as problem-solving therapy, acupuncture, music therapy (MT), exercise therapy, and motivational interviewing can alleviate depressive symptoms (14). However, evidence-based recommendations regarding the most effective NPIs for improving PSD are currently lacking. Therefore, it is crucial to identify appropriate NPIs that can effectively reduce PSD. Network meta-analysis (NMA), also known as a meta-analysis of mixed or multiple treatment comparisons (20), compares the impact of various NPIs on PSD by estimating both direct and indirect comparisons. Although a previously published NMA has been identified, it only reported the effects of pharmacological treatments and did not investigate NPIs further (21). Hence, the objective of this study was to conduct an NMA of relevant randomized controlled trials (RCTs) to compare the effects of different NPIs on PSD. The results of this study are essential for formulating clinical practice guidelines and recommending optimal intervention strategies to improve PSD.

## Methods

This NMA was designed based on the guidelines of the Preferred Reporting Items of Systematic Review and Network Meta-Analysis (22) and registered in the PROSPERO database (CRD42024501101).

### Search strategies

Searches for RCTs on PSD published up to December 2023 were conducted using databases such as PubMed, Web of Science, Embase, the Cochrane Library, China National Knowledge Infrastructure (VIP), and Wanfang. The search involved a combination of participants and free words. The search strategy is described in Appendix 1.

### Study selection

YL and LG were selected as independent reviewers to screen the titles and abstracts of the retrieved literature using search strategies to identify studies that met the inclusion criteria. In cases of disagreement, checks and discussions were performed by Qd D to reach a consensus. Data were deduplicated using EndNote (23). A full-text assessment of the potentially eligible studies was conducted based on the inclusion and exclusion criteria. Any differences between the reviewers were resolved through discussion, and the EndNote software was used to manage this phase.

### Inclusion criteria

The inclusion and exclusion criteria were based on the PICOS standards. Table 1 lists the specific inclusion and exclusion criteria.

**Table 1:** Inclusion and exclusion criteria.

Category	Inclusion criteria	Exclusion criteria
Population	(1) Age > 18 years, with a diagnosis of stroke based on computer tomography, magnetic resonance imaging, or clinical criteria; (2) depression symptoms clearly diagnosed according to the HAMD	Severe complications
Interventions	BDJ, AAMT, CBT, EMT, ACA, CBTA, NMES, TEPP, MTP, NCA, ACMT, MBSR, AAAS, ACT, VR, MUT	
Comparisons	COT	
Outcomes		
Study	RCT; published in English or Chinese	

AAAS, Acupoint acupuncture + auricular sticking; AAMT, Acupoint acupuncture + music therapy; ACA, Acupoint acupuncture; ACMT, Acupressure + music therapy; ACT, Acceptance and commitment therapy; BDJ, Baduanjin; CBT, Cognitive behavioral therapy; CBTA, Cognitive behavioral therapy + acupoint acupuncture; COT, Conventional therapy; EMT, Empathy technique; HAMD, Hamilton Depression Scale; MBSR, Mindfulness-based stress reduction; MUT, Music therapy; MTP, Music therapy + positive psychological intervention; NCA, Narrative care + acupressure; NMES, Neuromuscular electrical stimulation; TEPP, Team positive psychotherapy; VR, Virtual reality technology. RCT, randomized controlled trial.

### Risk-of-bias assessment

Two reviewers (LG and XM) independently assessed the risk of bias, and a third reviewer adjudicated using Cochrane collaboration tools, such as sequence generation, assignment hiding, blinding, incomplete outcome data, nonselective outcome reporting, and other sources of bias (24). Each criterion was considered as having a low, unclear, or high risk of bias (25).

### Data extraction

The following data were independently extracted from the reviewers: the first author, publication year, country, sample size, and outcome indicators. Data are expressed as mean ± standard deviation (SD).

### Data analysis

The “Netmeta” package in R-4.2.1 software was used for NMA. Network plots were generated using the STATA 15.1 “network plot” features to describe and present various forms of motion. Nodes were used to represent various interventions, and edges were used to depict favorable intervention comparisons. Inconsistencies between direct and indirect comparisons were evaluated using the node segmentation method (26). Combined estimates and 95% confidence intervals (95% CIs) were computed using a random-effects network element analysis. In studies in which the same measurement unit was of interest, the mean difference (MD) was considered a treatment effect when analyzing the results or evaluating the standardized MD (SMD). Different exercise treatments were compared using a pairwise random-effects meta-analysis. The heterogeneity of all pair-to-pair comparisons was evaluated using the *I*^2^ statistic, and publication bias was evaluated using the *p*-value of Egger’s test. Publication bias and secondary study effects, analyzed using the results of more than a dozen reported studies, were identified using funnel plots.

## Results

### Literature selection

**Figure 1:**
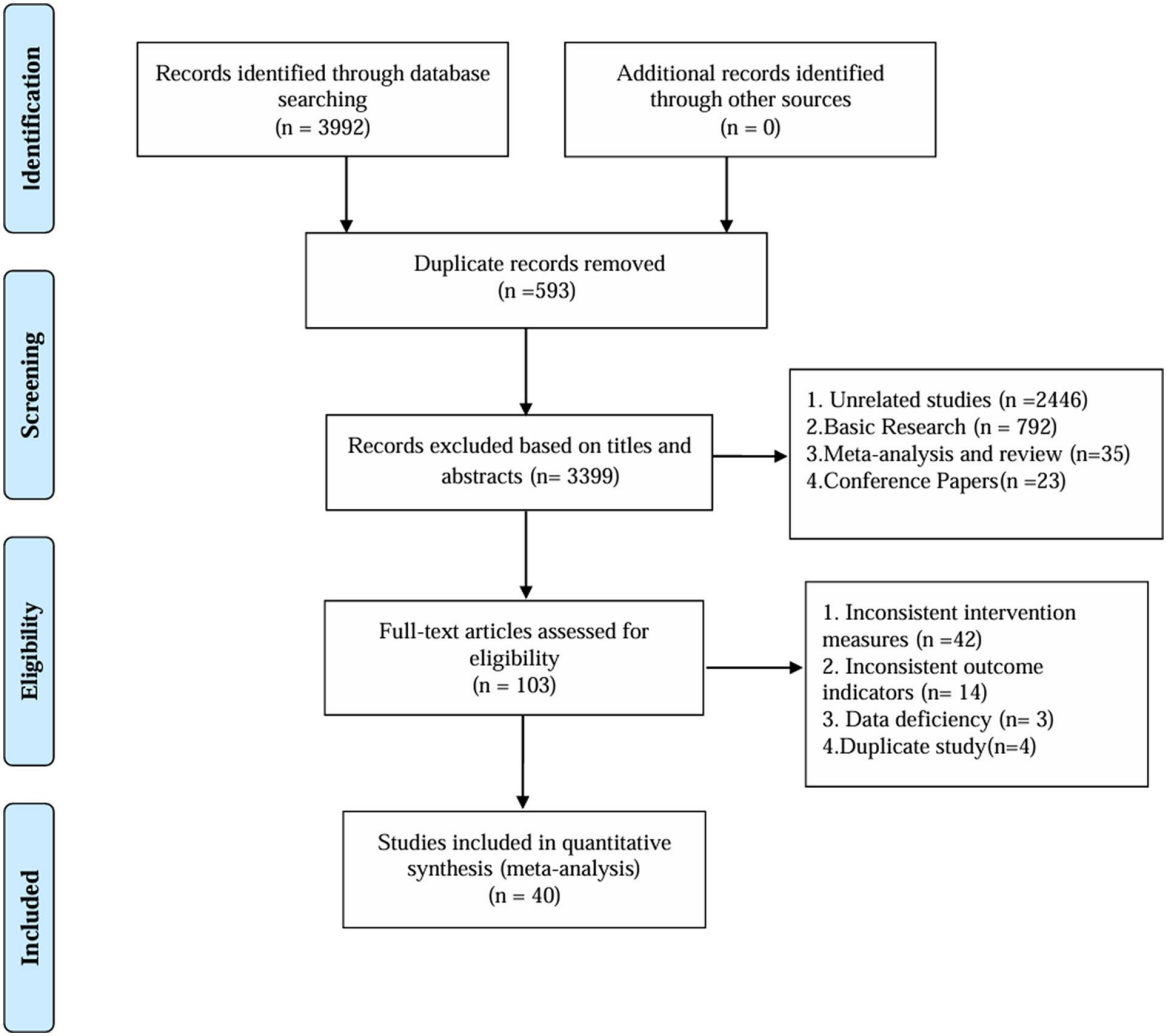
Flow of trials through the review.

After conducting the literature search, 3,992 articles were identified. After removing duplicate records, 3,399 articles remained for further analysis. Among the remaining 103 records, 63 were excluded because of inconsistent intervention measures (42 records), inconsistent outcome indicators (14 records), data deficiencies (3 records), and duplicate studies (4 records). Ultimately, 40 (27–66) studies were included. Figure 1 shows a flowchart of the study.

### Study and participant characteristics

**Figure 2:**
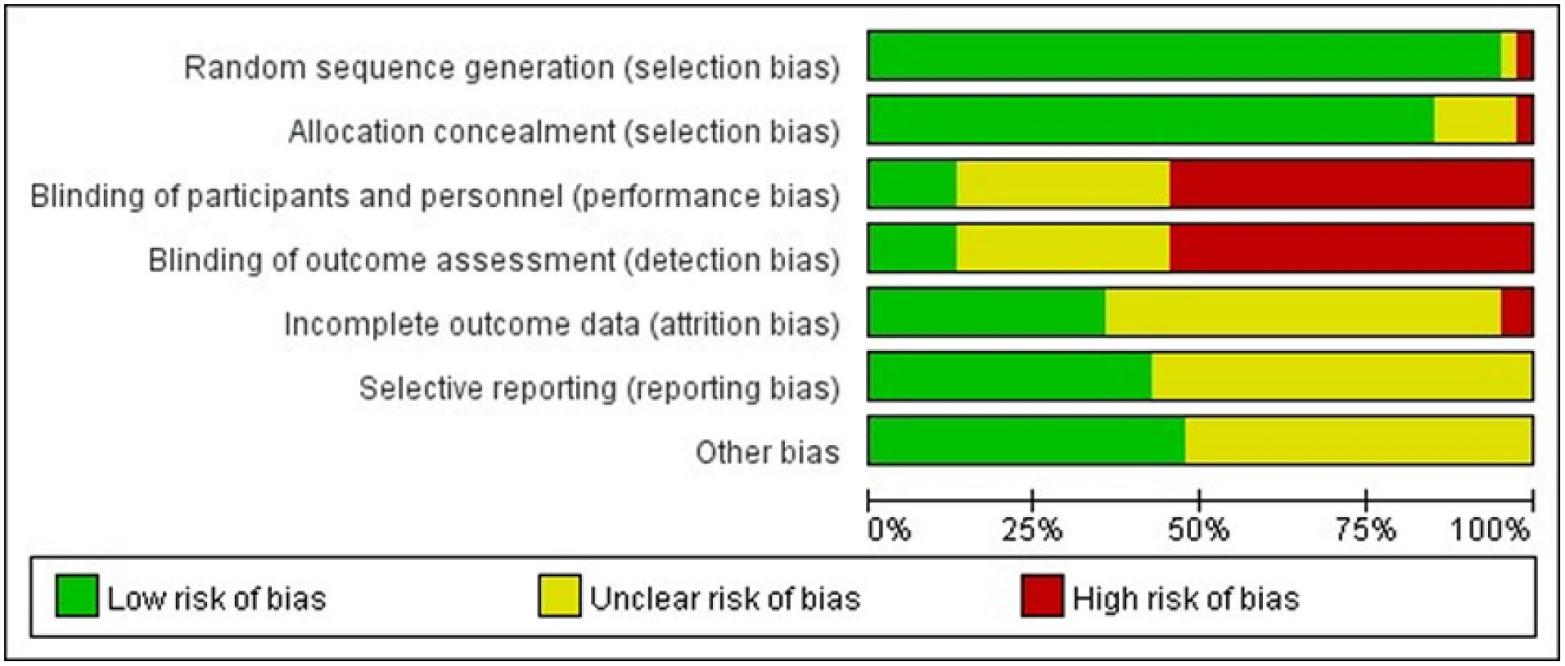
Percentage of studies examining the efficacy of NPIs in patients with PSD with low, unclear, and high risk of bias for each feature of the Cochrane Risk-of-Bias Tool.

Studies comparing the effects of 17 NPIs in patients with PSD published between 2006 and 2022 were included. A total of 3,225 patients were included in the selected studies. Among these studies, 40 reported the Hamilton Depression Scale (HAMD), and eight reported the Pittsburgh Sleep Quality Index (PSQI). The participants had an average age of 31–72 years. Table 2 presents the characteristics of the included studies and participants. The risk-of-bias assessment for each study is presented in Appendix 2, and Figure 2 presents the aggregated data.

**Table 2:** General characteristics of all included studies.

Name	Years	Country	Group	Age	Sample size	Intervention frequency/Intervention time	Outcomes
Liu (27)	2021	China	BDJ/COT	57.58 ± 5.71/56.85 ± 7.47	30/30	3 times/week, 45 min/time	HAMD/PSQI
Zhang (28)	2013	China	AAMT/COT	62.5/61.5	30/30	5 times/week, 30 min/time	HAMD
Li (29)	2021	China	CBT/COT	32.56 ± 3.06/31.97 ± 3.18	45/45	7 times/week, 30 min/time	HAMD
Wang (30)	2006	China	MUT/COT	<70	30/30	60 min/time	HAMD
Zhou (31)	2016	China	EMT/COT	63.1 ± 8.7/62.6 ± 8.2	60/60	NA	HAMD
Liu (32)	2021	China	AAMT/ACA	58.18 ± 5.25/57.76 ± 6.02	20/20	5 times/week	HAMD
Du (33)	2017	China	CBT/COT	71.11 ± 6.86/70.08 ± 6.81	45/44	NA	HAMD
Nie (34)	2020	China	CBT/COT	66.86 ± 3.40/67.60 ± 3.50	45/45	7 times/week, 30 min/time	HAMD/PSQI
Li (35)	2015	China	CBT/COT	55.84 ± 6.18/56.12 ± 5.36	60/60	2–3 days/time, 8 weeks	HAMD
Liu (36)	2016	China	CBT/COT	55.67 ± 5.52/55.64 ± 5.51	49/48	NA	HAMD
Fang (37)	2020	China	CBTA/COT	46.5 ± 15.7/47.6 ± 13.4	31/31	5 times/week, 30 min/time	HAMD
Huang (38)	2012	China	NMES/COT	68.32 ± 11.61/67.12 ± 12.37	41/41	1 time/day, 1 month	HAMD
Zhang (39)	2016	China	ACMT/COT	67.1 ± 10.6/63.2 ± 8.2	30/30	Once a day	HAMD
Li (40)	2020	China	TEPP/COT	67.04 ± 3.33/66.89 ± 3.45	54/53	8 weeks	HAMD
Zhang (41)	2018	China	AAMT/MUT/ACT	49.23 ± 8.14/49.98 ± 7.60/50.02 ± 7.87	21/21/21	Once a day	HAMD
Sun (42)	2020	China	MTP/COT	54 ± 5/53 ± 6	45/45	2 times/week, 10–20 min/time	HAMD
Wang (43)	2018	China	AAMT/ACA	59.3/59.3	40/40	1 time/day	HAMD/PSQI
Lin (44)	2016	China	ACA/AAMT/COT	72.93 ± 10.37/69.66 ± 10.41/68.80 ± 11.53	30/30/32	2 times/day	HAMD
Wang (45)	2019	China	ACA/MUT/AAMT	48.56 ± 7.82/49.53 ± 7.23/50.05 ± 6.89	30/30/30	2 times/week	HAMD/PSQI
Wang (46)	2022	China	CBT/MUT	58.97 ± 8.89/59.60 ± 8.35	35/36	2 times/week	HAMD
Rao (47)	2021	China	NCA/COT	56.32 ± 12.37/54.88 ± 13.03	41/41	2–3 times/week, 30–40 min/time	HAMD/PSQI
Li (48)	2019	China	ACMT/COT	64.58 ± 18.37/65.26 ± 17.62	63/63	2 times/day	HAMD/PSQI
Cui (49)	2007	China	MUT/COT	68.5 ± 3.2	29/29	2 times/week, 20–30 min/time	HAMD
Huang (50)	2018	China	AAMT/ACA	44.7 ± 7.1/45.9 ± 7.6	31/31	1 times/week, 30 min/time	HAMD
Lu (51)	2012	China	MUT/COT	62.5/61.5	48/50	20 min/time	HAMD
Weng (52)	2012	China	MUT/COT	60.1 ± 7.8/59.3 ± 8.5	30/30	2 times/day	HAMD
Zhu (53)	2010	China	MUT/COT	58.7 ± 9.3/59.4 ± 8.6	40/40	60 min/time	HAMD
Liu (54)	2016	China	MUT/COT	60.5 ± 12.7/61.1 ± 8.19	30/30	30 min/time	HAMD
Wang (55)	2017	China	CBT/COT	70.1 ± 6.86/69.5 ± 9.34	60/60	2 times/week, 30 min/time	HAMD
Xiao (56)	2011	China	AAMT/COT	62.5/61.5	57/56	5 times/week	HAMD
Liu (57)	2021	China	AAMT/COT	53 ± 7/52 ± 5	29/32	5 days/week	HAMD/PSQI
Yang (58)	2016	China	MUT/COT	62.81 ± 6.99/61.91 ± 7.76	69/68	5 times/week, 30 min/time	HAMD/PSQI
Pei (59)	2020	China	MUT/COT	67.33 ± 5.94/67.30 ± 5.73	60/60	1 time/day, 30 min/time	HAMD
Zhang (60)	2017	China	AAAS/COT	59 ± 9/58 ± 8	30/30	3 times/week	HAMD
Chen (61)	2018	China	ACA/COT	51.63 ± 1.63/50.40 ± 1.71	30/30	6 times/week	HAMD
Xu (62)	2015	China	MBSR/COT	56.59 ± 7.32/58.23 ± 6.55	34/34	8 weeks	HAMD
Xue (63)	2020	China	MBSR/COT	56.59 ± 7.32/58.23 ± 6.55	39/39	6 weeks	HAMD
Shin (64)	2015	Korea	VR/COT	53.3 ± 11.8/54.6 ± 13.4	16/16	6 day/week	HAMD
Niu (65)	2021	China	ACT/COT	61.5 ± 11.5/64.8 ± 12.1	49/48	2 weeks	HAMD
Maier (66)	2020	Spain	VR/COT	63.63 ± 6.73/67.21 ± 6.45	16/14	30 min/time, 6 weeks	HAMD

AAAS, Acupoint acupuncture + auricular sticking; AAMT, Acupoint acupuncture + music therapy; ACA, Acupoint acupuncture; ACMT, Acupressure + music therapy; ACT, Acceptance and commitment therapy; BDJ, Baduanjin; CBT, Cognitive behavioral therapy; CBTA, Cognitive behavioral therapy + acupoint acupuncture; COT, Conventional therapy; EMT, Empathy technique; MBSR, Mindfulness-based stress reduction; MUT, Music therapy; MTP, Music therapy + positive psychological intervention; NCA, Narrative care + acupressure; NMES, Neuromuscular electrical stimulation; TEPP, Team positive psychotherapy; VR, Virtual reality technology. RCT, randomized controlled trial; PSQI, Pittsburgh Sleep Quality Index; HAMD, Hamilton Depression Scale. Each intervention is defined in Appendix 6.

### Outcomes

**Figure 3:**
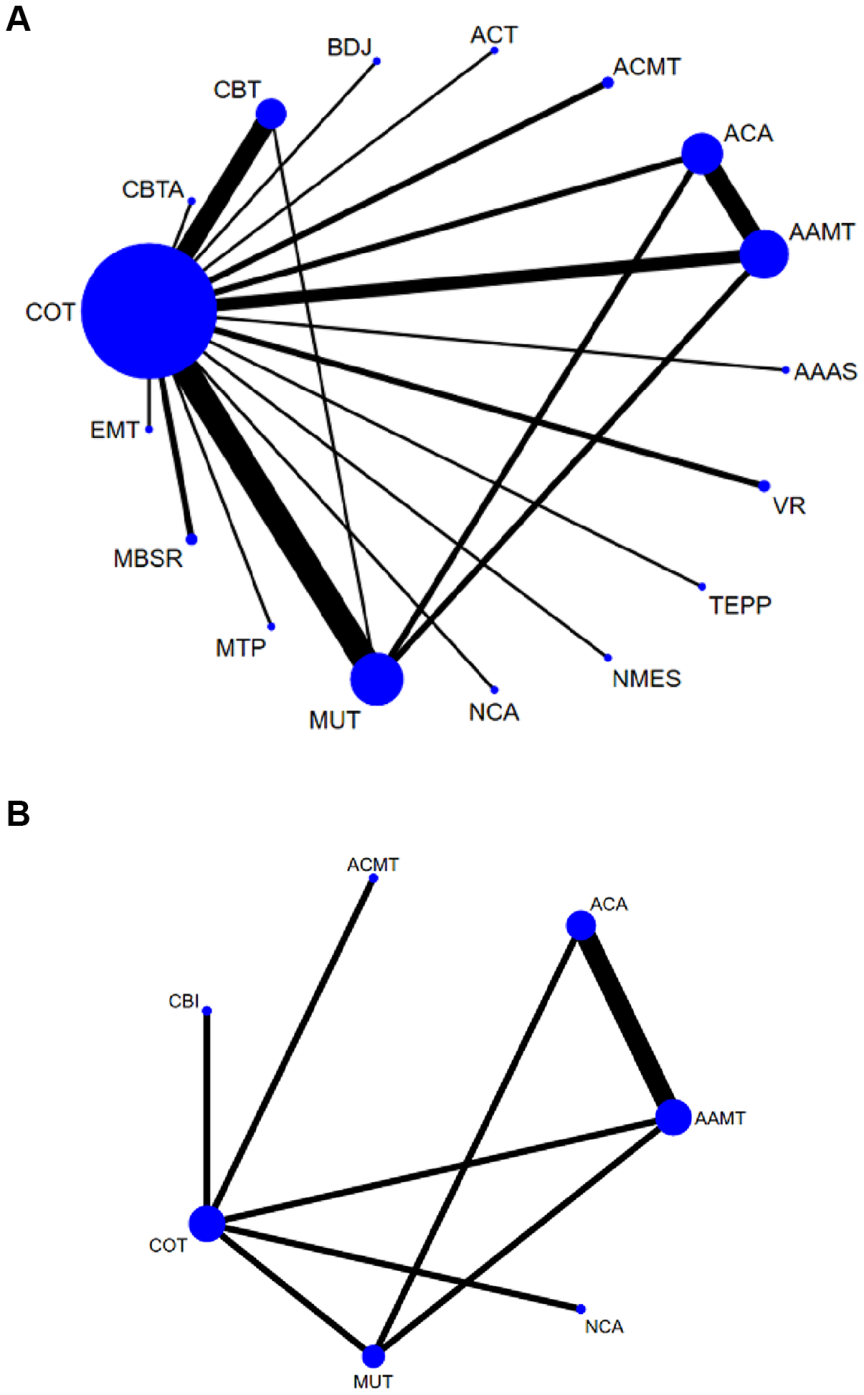
Network plots: the size of the nodes represents the number of times the exercise appears in any comparison of that treatment, and the width of the edges represents the total sample size in the comparisons it connects. AAAS, Acupoint acupuncture + auricular sticking; AAMT, Acupoint acupuncture + music therapy; ACA, Acupoint acupuncture; ACMT, Acupressure + music therapy; ACT, Acceptance and commitment therapy; BDJ, Baduanjin; CBT, Cognitive behavioral therapy; CBTA, Cognitive behavioral therapy + acupoint acupuncture; COT, Conventional therapy; EMT, Empathy technique; MBSR, Mindfulness-based stress reduction; MUT, Music therapy; MTP, Music therapy + positive psychological intervention; NCA, Narrative care + acupressure; NMES, Neuromuscular electrical stimulation; TEPP, Team positive psychotherapy; VR, Virtual reality technology.

HAMD: a total of 40 (27–66) studies, involving 3,225 participants, assessed HAMD. Seventeen interventions were included in the NMA (Figure 3A): Conventional therapy (COT), Cognitive behavioral therapy (CBT), Baduanjin (BDJ), Acceptance and commitment therapy (ACT), Acupressure + music therapy (ACMT), Acupoint acupuncture (ACA), Cognitive behavioral therapy + acupoint acupuncture (CBTA), Acupoint acupuncture + music therapy (AAMT), Acupoint acupuncture + auricular sticking(AAAS), Team positive psychotherapy (TEPP), Neuromuscular electrical stimulation(NMES), Narrative care + acupressure (NCA), Music therapy (MUT), Music therapy + positive psychological intervention (MTP), Mindfulness-based stress reduction (MBSR), and Empathy technique (EMT).

**Figure 4:**
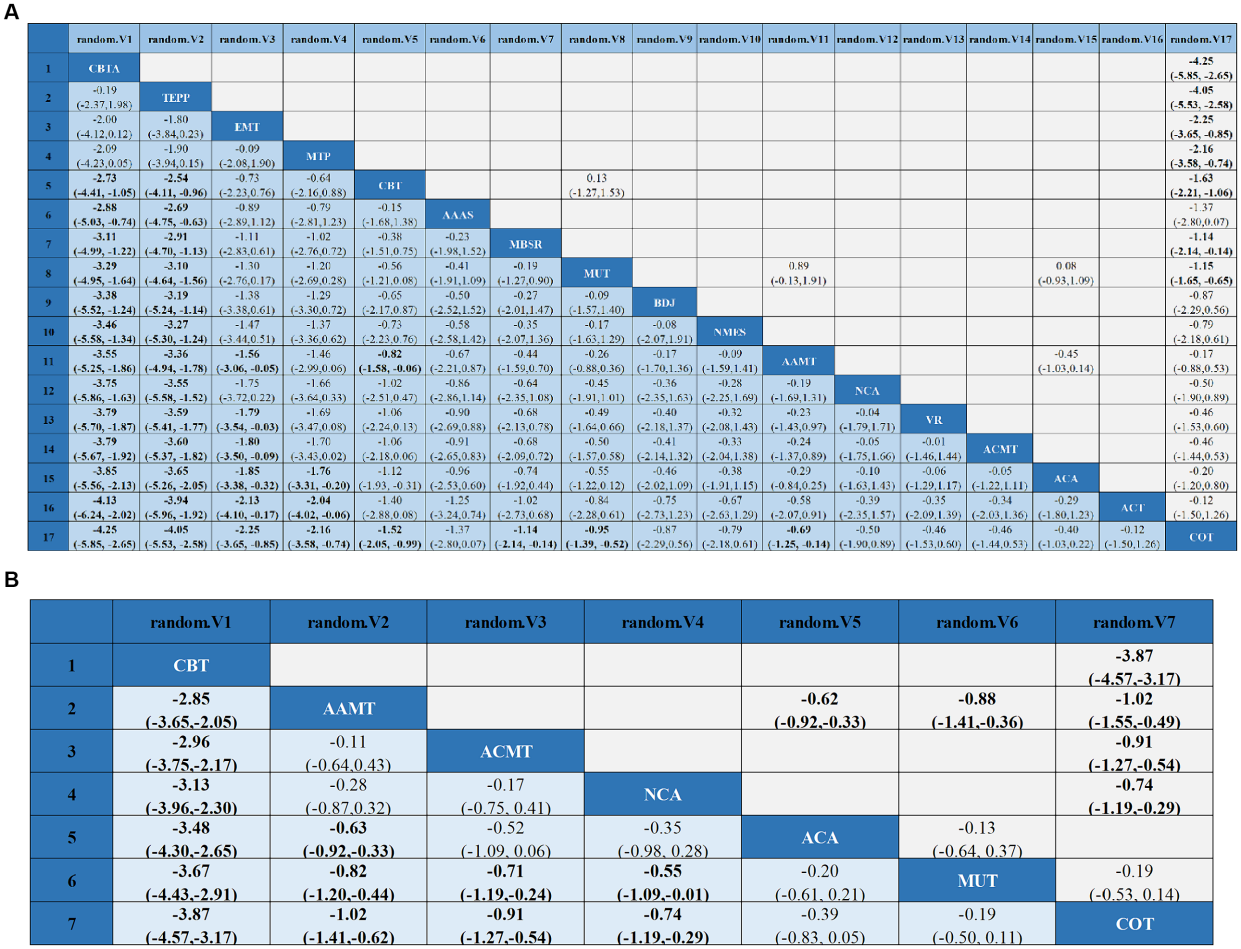
League tables of outcome analyses: data are mean differences and 95% credibility intervals for continuous data. AAAS, Acupoint acupuncture + auricular sticking; AAMT, Acupoint acupuncture + music therapy; ACA, Acupoint acupuncture; ACMT, Acupressure + music therapy; ACT, Acceptance and commitment therapy; BDJ, Baduanjin; CBT, Cognitive behavioral therapy; CBTA, Cognitive behavioral therapy + acupoint acupuncture; COT, Conventional therapy; EMT, Empathy technique; MBSR, Mindfulness-based stress reduction; MUT, Music therapy; MTP, Music therapy + positive psychological intervention; NCA, Narrative care + acupressure; NMES, Neuromuscular electrical stimulation; TEPP, Team positive psychotherapy; VR, Virtual reality technology.

Compared with COT, CBTA (MD, −4.25; 95% CI, −5.85 to −2.65), TEPP (MD, −4.05; 95% CI, −5.53 to −2.58), EMT (MD, −2.25; 95% CI, −3.65 to −0.85), CBT (MD, −1.52; 95% CI, −2.05 to −0.99), MBSR (MD, −1.14; 95% CI, −2.14 to −0.14), MUT (MD, −0.95; 95% CI, −1.39 to −0.52), and AAMT (MD, −0.69; 95% CI, −1.25 to −0.14) reported superior PSD improvement. Additionally, CBTA was more conducive to improving PSD than CBT (MD, −2.73; 95% CI, −4.41 to −1.05), AAAS (MD, −2.88; 95% CI, −5.03 to −0.74), MBSR (MD, −3.11; 95% CI, −4.99 to −1.22), MT (MD, −3.29; 95% CI, −4.95 to −1.64), BDJ (MD, −3.38; 95% CI, −5.52 to −1.24), NSES(MD, −3.46; 95% CI, −5.58 to −1.34), AAMT (MD, −3.55; 95% CI, −5.25 to −1.86), NCA (MD, −3.75; 95% CI, −5.86 to −1.63), VR (MD, −3.79; 95% CI, −5.70 to −1.87), ACMT (MD, −3.79; 95% CI, −5.67 to −1.92), ACA (MD, −3.85; 95% CI, −5.56 to −2.13), ACT (MD, −4.13; 95% CI, 6.24 to −2.02) (Figure 4A). Comparison of the adjusted funnel plots did not provide evidence of significant publication bias, as confirmed by Egger’s test (*p* = 0.959) (Appendix 3.1). Heterogeneity, intransitivity, and inconsistencies in the NMAs were also evaluated (Appendix 4). Furthermore, direct comparisons of the HAMD scores were performed (Appendix 5.1).

Sleep quality: In 8 (27, 34, 43, 45, 47, 48, 57, 58) studies, the PSQI was assessed in 726 participants. Seven interventions were included in the NMA (Figure 3B): Conventional therapy (COT), Narrative care + acupressure (NCA), Cognitive behavioral therapy (CBT), Acupressure + music therapy (ACMT), Acupoint acupuncture (ACA), Acupoint acupuncture + music therapy (AAMT), Music therapy (MUT). CBT (MD, −3.67; 95% CI, −4.43 to −2.91), AAMT (MD, −0.82; 95% CI, −1.20 to −0.44), ACMT (MD, −0.71; 95% CI, −1.19 to −0.24), NCA (MD, −0.55; 95% CI, −1.09 to −0.01) demonstrated superior PSQI improvement compared with MUT. Furthermore, CBT (MD, −3.87; 95% CI, −4.57 to −3.17), AAMT (MD, −1.02; 95% CI, −1.41 to −0.62), ACMT (MD, −0.91; 95% CI, −1.27 to −0.54), and NCA (MD, −0.74; 95% CI, −1.19 to −0.29) demonstrated superior PSQI improvement compared with COT (Figure 4B). A comparison of the adjusted funnel plot did not provide evidence of significant publication bias, as confirmed by the Egger’s test (*p* = 0.356) (Appendix 3.2). Heterogeneity, inaccessibility, and inconsistencies in the NMAs were evaluated (Appendix 4). In addition, direct comparisons of the PSQI scores were evaluated (Appendix 5.2).

## Discussion

Depression is the most common neuropsychiatric complication after cerebrovascular accidents, affecting approximately one-third of stroke survivors. The core symptom cluster includes low mood, emotional blunting, fatigue, insomnia, feelings of worthlessness, and even suicidal ideation (7, 9, 67, 68). The use of pharmacotherapy to treat PSD can lead to adverse reactions, symptom withdrawal, and drug resistance. Therefore, there is an urgent need to seek alternative complementary therapies (69). NPIs have fewer adverse effects than drug therapies and have become a popular treatment option for PSD. However, current NPIs comprise various treatment modalities. In this study, we obtained 40 articles and analyzed the effects of 17 types of NPIs on PSD to determine which intervention could effectively improve PSD occurrence, alleviate sleep quality, and improve quality of life.

The findings of this study indicate that Cognitive behavioral therapy + acupoint acupuncture (CBTA), Team positive psychotherapy (TEPP), Empathy technique (EMT), Cognitive behavioral therapy (CBT), Neuromuscular electrical stimulation(NMES), Music therapy (MUT), and Acupoint acupuncture + music therapy (AAMT) are more effective than COT in improving depression in patients with PSD. The pathogenesis of PSD remains unclear, although some studies have suggested that it may be associated with the blockade of noradrenergic and serotonergic neuronal pathways caused by stroke (70). Other studies have indicated that the etiology of PSD is multifactorial and includes biological, psychological, and social influences. CBT aims to improve PSD symptoms and enhance patients’ quality of life. These interventions involve encouraging patients to express their emotions, helping them recognize negative emotions and their consequences, correcting negative habitual thoughts and maladaptive cognitions, and implementing stimulus control therapy to enhance their responsiveness to sleep, alleviate depression, and improve their overall well-being. Acupuncture at specific acupoints is a traditional treatment method used in China that has various therapeutic effects, including alleviating liver and depression symptoms, calming the heart and mind, promoting the circulation of qi and blood, and modulating the expression levels of brain-derived neurotrophic factor and 5-hydroxytryptamine. These effects contribute to the improvement of depressive symptoms and enhancement of daily life functioning (71). The anterior cingulate gyrus (ACC) has extensive fibrous connections with many cortical and subcortical structures and is involved in the regulation of emotion and other functions. ACC can be significantly activated upon receiving negative emotional stimuli; thus, it is regarded as a key structure in the pathogenesis of depression (72). fMRI results have shown that the whole-brain connectivity of multiple regions, such as the medial and lateral prefrontal cortex, was reduced in depressed patients compared with that in healthy volunteers (73), and functional connectivity between the anterior dorsal cingulate cortex and dorsolateral frontal lobe was enhanced (74). A previous study (75) has shown that acupuncture at the Baihui point can regulate the default mode network in patients with depression and induce enhanced functional connections between the posterior central gyrus, prefrontal cortex, and bilateral ACC. In another study (76), stimulating the transcutaneous vagus nerve (tVNS) significantly reduced the HAMD scores in patients with depression. fMRI results showed that tVNS significantly regulated the resting-state functional connections in the frontal amygdala of patients with depression. In traditional Chinese medicine, “depression” belongs to the category of “depression syndrome,” its cause is emotional injury, and its incidence is related to the dysfunction of the heart, liver, and kidney (77). Kehua found that the two channels of the Du pulse and liver of the Jueyin of the foot were combined and used to stimulate Yang Qi and inject blood essence and fluid, which significantly prevented cerebral psychosis (78). Furthermore, acupuncture can significantly enhance neurological function and activities of daily living in patients with stroke. The efficacy of acupuncture is comparable to or even superior to that of drug therapy, with fewer adverse reactions, higher safety, and better patient compliance (79, 80). Acupuncture has a persistent effect; repeated acupuncture has a cumulative effect, and the therapeutic effect can be enhanced by multiple treatments. Repeated treatment helps maintain and enhance initial improvement. However, one study reported that the effects of acupuncture diminish over time (81). Choosing the appropriate acupuncture course is important to ensure the sustained effect of acupuncture and consolidate its curative effect (82). Group psychotherapy has been widely used and recognized as a clinical treatment modality (83). By incorporating components such as rehabilitation discussions and confidence-enhancing exercises, group psychotherapy fosters a sense of team spirit and cohesion among patients. In turn, this encourages the development of an optimistic outlook towards life and active coping style for the disease. Group games are primarily employed to shift attention and promote the recognition of the beauty of life. However, meditation and relaxation training can soothe inner turmoil, enhance patient happiness, and reduce depressive symptoms.

The findings of this study indicate that Cognitive behavioral therapy (CBT), Acupoint acupuncture + music therapy (AAMT), Acupressure + music therapy (ACMT), and Narrative care + acupressure (NCA) are more effective than Conventional therapy (COT) in improving sleep quality in patients with PSD. CBT has demonstrated an efficacy comparable to that of medication in the treatment of moderate-to-severe depression in the general population. This therapeutic approach aids patients in regulating their emotions, attaining optimal activity and function levels, and maintaining realistic and optimistic thinking patterns (84). Furthermore, CBT addresses maladaptive cognitive structures by restructuring erroneous thought processes and implementing behavioral interventions. Specifically, cognitive therapy encompasses the provision of sleep education, correction of misconceptions regarding sleep, and assistance in establishing reasonable expectations for enhancing sleep quality. The preautonomic neurons of the hypothalamic paraventricular nucleus are the main targets of the SCN, which affects the motor nucleus of the hypothalamic vagus nerve and preganglionic motor neurons of the spinal cord (85). This allows the SCN to affect sympathetic and parasympathetic outputs in all organs (86). Different sympathetic nerves project neurons to different organs, thereby providing an anatomical basis for the control of different organs (86). The SCN controls the circadian rhythm of melatonin synthesis in the pineal gland through a multisynaptic pathway, preautonomic neurons of the PVN, parasympathetic neurons of the spinal cord, and norepinephrinergic neurons in the superior cervical ganglion. Norepinephrine is a sympathetic neurotransmitter with an obvious circadian rhythm that activates the internal circadian rhythm of cardiomyocytes in a serum-free manner (87). The American Medical Association guidelines recommend CBT as the first-line treatment for insomnia (88). The main mechanism of CBT is to promote rapid and effective neural guidance at the thalamus level to initiate sleep, reduce the activity of the whole sympathetic nervous system of patients, and weaken cognitive psychological “arousal,” so as to assist in inducing sleep (89, 90). However, the mechanism of action at the basic level of physiological anatomy, circadian rhythm changes, and biochemical changes remains unclear and requires further research (91). In addition, studies have found that vagus nerve stimulation can significantly activate the nucleus of the solitary tract; project fibers to the parabrachial nucleus, locus coeruleus, raphe nucleus, reticular structure, thalamus, and other central sleep structures; and participate in sleep regulation of sleep (92). CBT can stimulate the reticular structure of the brain and regulate the central nervous system, which is conducive to improving sleep disorders in patients (93). CBT is effective in improving sleep efficiency and reducing the number of waking times after falling asleep and the latency to fall asleep (94).

AAMT is a noninvasive and well-accepted treatment approach that involves the use of music to stimulate the central nervous system, induce a state of calmness, alleviate pain, and reduce negative emotions, thereby improving sleep quality in patients with PSD (69). MT, a noninvasive natural therapy, offers a safe and cost-effective option worthy of promotion (69). ACA has shown potential efficacy in treating sleep disorders; however, the underlying mechanisms have not yet been fully elucidated. Research indicates that ACA modulates the activity of neurotransmitters and hormones involved in sleep regulation, including melatonin, serotonin, and gamma-aminobutyric acid (GABA) (95). Additionally, it can regulate the autonomic nervous system by reducing sympathetic nerve activity and increasing parasympathetic nerve activity, leading to a more relaxed state conducive to sleep.

## Study strengths and limitations

This review has several advantages. First, it provides NMAs that directly and indirectly compare various intervention measures. Moreover, more accurate intervention measures are included and carefully categorized into 17 different interventions, with each intervention being clearly defined. Second, the effects of various interventions on PSD and PSQI were studied, and other intervention measures were analyzed. Thus, the findings of this study serve as a reference.

However, this study has certain limitations. First, the duration, intensity, and frequency of interventions were not considered. Second, the implementation quality of blinding in the included studies was not high, and outcome measures were all subjective indicators. An explanation of the biological parameters should be added. Third, only Chinese and English studies were included, which may have resulted in heterogeneity. Fourth, all studies were small-scale; therefore, future large-scale studies are recommended. Fifth, some of the studies included in this study had a high risk of bias due to the lack of blinding, which will have a certain impact on the results of this study. Finally, this study did not consider the impact of factors such as severity of depression, patients’ medication treatment, or other factors on PSD, which may have a particular influence on the results.

## Conclusion

Evidence from systematic reviews and meta-analyses recommends that CBT and ACA improve depression in patients with PSD. CBT should be used to improve sleep in patients. The results of this study are limited, and future studies should include more high-quality studies to further validate the findings and select appropriate interventions based on the circumstances of stroke patients.

## Data availability statement

The original contributions presented in the study are included in the article/supplementary material, further inquiries can be directed to the corresponding author.

## Author contributions

YL: Conceptualization, Data curation, Formal analysis, Methodology, Software, Writing – original draft. YW: Writing – review & editing, Supervision, Resources. LG: Data curation, Software, Writing – original draft. XM: Data curation, Resources, Software, Writing – original draft. QD: Data curation, Resources, Writing – original draft.

## References

[1] Feigin VL Brainin M Norrving B Martins S Sacco RL Hacke W . World stroke organization (WSO): global stroke fact sheet 2022. Int J Stroke (2022) 17:18–29. doi: 10.1177/17474930211065917, PMID: 34986727

[2] Kapoor A Lanctôt KL Bayley M Kiss A Herrmann N Murray BJ . "Good outcome" Isn't good enough: cognitive impairment, depressive symptoms, and social restrictions in physically recovered stroke patients. Stroke (2017) 48:1688–90. doi: 10.1161/STROKEAHA.117.016728, PMID: 28438907

[3] Cai W Mueller C Li YJ Shen WD Stewart R. Post stroke depression and risk of stroke recurrence and mortality: a systematic review and meta-analysis. Ageing Res Rev (2019) 50:102–9. doi: 10.1016/j.arr.2019.01.013, PMID: 30711712

[4] Das J Rajanikant GK. Post stroke depression: the sequelae of cerebral stroke. Neurosci Biobehav Rev (2018) 90:104–14. doi: 10.1016/j.neubiorev.2018.04.005, PMID: 29656030

[5] Ahmed ZM Khalil MF Kohail AM Eldesouky IF Elkady A Shuaib A. The prevalence and predictors of post-stroke depression and anxiety during COVID-19 pandemic. J Stroke Cerebrovasc Dis (2020) 29:105315. doi: 10.1016/j.jstrokecerebrovasdis.2020.105315, PMID: 32958396 PMC7834239

[6] Schöttke H Giabbiconi CM. Post-stroke depression and post-stroke anxiety: prevalence and predictors. Int Psychogeriatr (2015) 27:1805–12. doi: 10.1017/S1041610215000988, PMID: 26178418

[7] He JR Zhang Y Lu WJ Liang HB Tu XQ Ma FY . Age-related frontal periventricular white matter Hyperintensities and miR-92a-3p are associated with early-onset post-stroke depression. Front Aging Neurosci (2017) 9:328. doi: 10.3389/fnagi.2017.00328 29051732 PMC5633610

[8] Robinson RG Spalletta G. Poststroke depression: a review. Can J Psychiatr (2010) 55:341–9. doi: 10.1177/070674371005500602, PMID: 20540828 PMC3647458

[9] Egorova N Cumming T Shirbin C Veldsman M Werden E Brodtmann A. Lower cognitive control network connectivity in stroke participants with depressive features. Transl Psychiatry (2018) 7:4. doi: 10.1038/s41398-017-0038-x, PMID: 29520018 PMC5843603

[10] Duan X Yao G Liu Z Cui R Yang W. Mechanisms of transcranial magnetic stimulation treating on post-stroke depression. Front Hum Neurosci (2018) 12:215. doi: 10.3389/fnhum.2018.00215 29899693 PMC5988869

[11] Miranda JJ Moscoso MG Toyama M Cavero V Diez-Canseco F Ovbiagele B. Role of mHealth in overcoming the occurrence of post-stroke depression. Acta Neurol Scand (2018) 137:12–9. doi: 10.1111/ane.12832, PMID: 28901543 PMC5716920

[12] Wang EY Meyer C Graham GD Whooley MA. Evaluating screening tests for depression in post-stroke older adults. J Geriatr Psychiatry Neurol (2018) 31:129–35. doi: 10.1177/0891988718778791, PMID: 29793370

[13] Villa RF Ferrari F Moretti A. Post-stroke depression: mechanisms and pharmacological treatment. Pharmacol Ther (2018) 184:131–44. doi: 10.1016/j.pharmthera.2017.11.005, PMID: 29128343

[14] Hadidi NN Huna Wagner RL Lindquist R. Nonpharmacological treatments for post-stroke depression: an integrative review of the literature. Res Gerontol Nurs (2017) 10:182–95. doi: 10.3928/19404921-20170524-02, PMID: 28556875

[15] Apóstolo J Bobrowicz-Campos E Rodrigues M Castro I Cardoso D. The effectiveness of non-pharmacological interventions in older adults with depressive disorders: a systematic review. Int J Nurs Stud (2016) 58:59–70. doi: 10.1016/j.ijnurstu.2016.02.006, PMID: 27087298

[16] Williams GO. Management of depression in the elderly. Prim Care (1989) 16:451–74. doi: 10.1016/S0095-4543(21)01100-3 2664841

[17] Lee Y Chen B Fong MWM Lee JM Nicol GE Lenze EJ . Effectiveness of non-pharmacological interventions for treating post-stroke depressive symptoms: systematic review and meta-analysis of randomized controlled trials. Top Stroke Rehabil (2021) 28:289–320. doi: 10.1080/10749357.2020.1803583, PMID: 32783504 PMC7878573

[18] Lett HS Davidson J Blumenthal JA. Nonpharmacologic treatments for depression in patients with coronary heart disease. Psychosom Med (2005) 67:S58–62. doi: 10.1097/01.psy.0000163453.24417.97 15953803

[19] Lökk J Delbari A. Management of depression in elderly stroke patients. Neuropsychiatr Dis Treat (2010) 6:539–49. doi: 10.2147/NDT.S7637, PMID: 20856917 PMC2938303

[20] Bafeta A Trinquart L Seror R Ravaud P. Reporting of results from network meta-analyses: methodological systematic review. BMJ (2014) 348:g1741. doi: 10.1136/bmj.g1741, PMID: 24618053 PMC3949412

[21] Zhang J Song Z Gui C Jiang G Cheng W You W . Treatments to post-stroke depression, which is more effective to HAMD improvement? Front Pharmacol (2022) 13:1035895. doi: 10.3389/fphar.2022.1035895, PMID: 36601053 PMC9806231

[22] Moher D Shamseer L Clarke M Ghersi D Liberati A Petticrew M . Preferred reporting items for systematic review and meta-analysis protocols (PRISMA-P) 2015 statement. Syst Rev (2015) 4:1. doi: 10.1186/2046-4053-4-1 25554246 PMC4320440

[23] Kali A Srirangaraj S. EndNote as document manager for summative assessment. J Postgrad Med (2016) 62:124–5. doi: 10.4103/0022-3859.174158, PMID: 26767973 PMC4944344

[24] Higgins JP Altman DG Gøtzsche PC Jüni P Moher D Oxman AD . The Cochrane Collaboration's tool for assessing risk of bias in randomised trials. BMJ (2011) 343:d5928. doi: 10.1136/bmj.d5928, PMID: 22008217 PMC3196245

[25] Jardim PSJ Rose CJ Ames HM Echavez JFM Van de Velde S Muller AE. Automating risk of bias assessment in systematic reviews: a real-time mixed methods comparison of human researchers to a machine learning system. BMC Med Res Methodol (2022) 22:167. doi: 10.1186/s12874-022-01649-y 35676632 PMC9174024

[26] Rücker G Schwarzer G. Ranking treatments in frequentist network meta-analysis works without resampling methods. BMC Med Res Methodol (2015) 15:58. doi: 10.1186/s12874-015-0060-8 26227148 PMC4521472

[27] Xiaoyu L Wenjie Z Jing L Xiao A Guangrong D. Influence of traditional exercise Baduanjin on post-stroke depression. Clinical Res TCM (2021) 13:86–8. doi: 10.3969/j.issn.1674-7860.2021.26.027

[28] Xue Z Wentao D Tianqing Q. Clinical observation on the efficacy and safety of electroacupuncture combined with Buddhist music in the treatment of post-stroke depression. Clin J Acupuncture (2013):13–5. doi: 10.3969/j.issn.1005-0779.2013.10.004

[29] Xujing L Yuehua L Xueqin L Lili T. Effects of cognitive-behavioral psychological care on post-stroke depression in young adults. Henan Med Res (2021) 30:4016–9. doi: 10.3969/j.issn.1004-437X.2021.21.055

[30] Ling W Wenli S Zhiying R Lihong C Limei F. Application of sensorial music therapy in rehabilitation of post-stroke depressed patients. Nurs Res (2006):2105–6. doi: 10.3969/j.issn.1009-6493.2006.23.021

[31] Yun Z Shixin W. Effects of empathy techniques on depression and cognitive function in stroke patients. Chin J Gerontol (2016) 36:855–6. doi: 10.3969/j.issn.1005-9202.2016.04.039

[32] Yan L He B Deng Z Li L Jianfei L Jian W . The effect of head needle-based music therapy on post-stroke depression in resting state functional magnetic resonance observation. Chin Rehabil Theory Practice (2021) 27:282–9. doi: 10.3969/j.issn.1006-9771.2021.03.006

[33] Lifeng D. Effects of cognitive intervention on post-stroke depression and ability of daily living in elderly patients. Chin J Pract Neurol Diseases (2017) 20:99–101. doi: 10.3969/j.issn.1673-5110.2017.17.033

[34] Beibei N Danyang Q Yali S Xiaozhen S. Effect of cognitive behavioral intervention on sleep quality in elderly patients with stroke depression. Int J Psychiatry (2020) 47:1049–1052+1056.

[35] Aiqin L Zhimin Z Yonghong Y Huijuan W. Effect of cognitive behavioral intervention on rehabilitation of patients with post-stroke depression. Chin Geriatr Health Med (2015) 13:125–6. doi: 10.3969/j.issn.1672-4860.2015.01.057

[36] Xiaoli L. Effect of cognitive behavioral intervention on improving depressive disorder and quality of life in stroke patients. Chin J Pract Neurol Diseases (2016) 19:111–2. doi: 10.3969/j.issn.1673-5110.2016.20.069

[37] Yingzi F Anqi C. Effect of cognitive behavioral therapy combined with electroacupuncture on post-stroke depression. Tianjin. Nursing (2020) 28:219–21. doi: 10.3969/j.issn.1006-9143.2020.02.031

[38] Aihua H Liuyi L Jing H Ping R. Effect of neuromuscular electrical stimulation on depression in convalescent stroke patients. J Adv Nurs (2012) 27:1479–80.

[39] Shunfeng Z Yan Z. Influence of head acupressure combined with music therapy on rehabilitation of post-stroke depressed patients. Traditional Mongolian Med (2016) 35:129. doi: 10.3969/j.issn.1006-0979.2016.14.117

[40] Meng L Yuanli G Aixia W Caixia Y. Effects of group positive psychotherapy on cognitive function and mood in elderly patients with post-stroke depression. Int J Psychiatry (2020) 47:812-814–21.

[41] Yao Z Jinglong S Chengjie L. Observation on the effect of five elements music therapy combined with acupuncture in the treatment of post-stroke depression. Tradit Mongolian Med (2018) 37:99–101. doi: 10.3969/j.issn.1006-0979.2018.07.065

[42] Ruili S Hongjin R Lixia H Guihua C Chrysanthemum I Runmei W . Application of five elements music therapy combined with positive psychological intervention in patients with post-ischemic stroke depression. Chin Med Clinic (2020) 20:2437–9. doi: 10.11655/zgywylc2020.14.068

[43] Min W Li L. Clinical observation of five elements music combined with acupuncture in the treatment of post-stroke depression. Shandong. J Chin Med (2018) 37:906–908+919. doi: 10.16295/j.cnki.0257-358x.2018.11.009

[44] Facai L Dehong H Yuhang Q Yihuang G Yunchuan W. Study on the efficacy and safety of five elements music in the treatment of post-stroke depression. Chin J Rehabil Med (2017) 32:1390–3. doi: 10.3969/j.issn.1001-1242.2017.12.014

[45] Ning W Jianzhong L Yi D Yan L Deng Z Li L . Observation on the curative effect of "five tones god" method in the treatment of post-stroke depression. J Rehabil Res Dev (2019) 29:44–8. doi: 10.3724/SP.J.1329.2019.06044

[46] Jian W Li L Wenyan Z Jianfei L Li L. Effect of five tones on psychological and sleep of patients with post-ischemic stroke depression. Nurs J (2022) 37:46–49+57. doi: 10.3870/j.issn.1001-4152.2022.06.046

[47] Lu R Li Z Biling Y Yili JC Huling L . Influence of narrative nursing combined with acupoint massage of traditional Chinese medicine on depression symptom group after stroke. Evid Based Nurs (2021) 7:125–8. doi: 10.12102/j.issn.2095-8668.2021.01.027

[48] Jingli L. Effect of acupressure combined with five elements music therapy on clinical symptoms and quality of life in patients with post-stroke depression and sleep disorders. Sichuan Tradit Chin Med (2019) 37:190–2.

[49] Jinhong C Na L. Effect of music relaxation therapy on 58 patients with post-stroke depression. Chin J Pract Neurol Diseases (2007):138–9. doi: 10.3969/j.issn.1673-5110.2007.03.093

[50] Lijun H Feng W Laiyong H. Therapeutic effect of music rehabilitation training combined with acupuncture on post-stroke depression. Chin J Rehabil Med (2018) 33:1447–50. doi: 10.3969/j.issn.1001-1242.2018.12.014

[51] Rong L Jun W Yanqiu M. The rehabilitation effect of music therapy on elderly patients with post-stroke depression. Southwest Natl Defense Med (2012) 22:405–6. doi: 10.3969/j.issn.1004-0188.2012.04.022

[52] Zheng WS. Intervention of music therapy on patients with post-stroke depression. Chin J Health Psychol (2012) 20:1542–4.

[53] Jianzhong Z Zhaoxin Z Heng Z. The rehabilitation effect of music therapy on patients with post-stroke depression. Rehabil China (2010) 25:437–8. doi: 10.3870/zgkf.2010.06.014

[54] Bo L. Study on the influence of music therapy on post-stroke depression. Electr J Cardiovascular Diseases Integr Chinese Western Med (2016) 4:117–8.

[55] Si W Shilie W Tai L. Effects of early cognitive behavioral therapy on depression and neurological function in elderly stroke patients. J Cardio-Cerebrovascular Diseases Integr Chin Western Med (2017) 15:2629–32. doi: 10.3969/j.issn.1672-1349.2017.20.039

[56] Zhanci X Zhouyi W Xia O Li C. Therapeutic effect of acupuncture combined with music therapy on post-stroke depression. J Cardio-Cerebrovascular Diseases Integr Chin Western Med (2011) 9:437–8. doi: 10.3969/j.issn.1672-1349.2011.04.031

[57] Honghua L Li T Qian L Keyi P Meilan L. Application of TCM five-tone therapy combined with acupoint pointer differentiation in patients with post-stroke depression. General Nursing (2021) 19:3398–401. doi: 10.12104/j.issn.1674-4748.2021.24.022

[58] Hongyan Y Xuegong F Hawwenjie YW Jing L Xiaolei W. Application of traditional Chinese medicine music therapy in patients with post-stroke depression. Nursing Practice Res (2016) 13:134–6. doi: 10.3969/j.issn.1672-9676.2016.14.061

[59] Lei P Ming X Yuqing Z Zhigang L Jifei S Zeping H. Observation on the curative effect of midday and noon flow timing five-tone therapy in the treatment of post-stroke depression. Guangxi Med (2020) 42:2721–4. doi: 10.11675/j.issn.0253-4304.2020.20.25

[60] Lin Z Yan Z Lim JS Yehui L Xuehui S Zhenguang L . Acupuncture combined with auricular point compression in the treatment of post-stroke depression: a randomized controlled study. Chin Acupunct Moxibustion (2017) 37:581–5. doi: 10.13703/j.0255-2930.2017.06.003 29231496

[61] Aiwen C Heng G Guantao W Jia L Weidong S. Effect of early acupuncture intervention on post-stroke depression: a randomized controlled study. Chin Acupunct Moxibustion (2018) 38:1141–4. doi: 10.13703/j.0255-2930.2018.11.001 30672192

[62] Jingwen X Birong L Xiaobo P Can H Jing L Yali Z . The effect of mindfulness-based stress reduction therapy on post-stroke depression. Mental Health Sichuan (2015) 28:523–5.

[63] Xiaoxia X Yanling H Shuyu L Zhen C Hui C Jinna W. Effects of mindfulness training on mental resilience, sleep and quality of life in patients with post-stroke depression. Chin J Health Psychol (2020) 28:1187–91. doi: 10.11886/j.issn.1007-3256.2015.06.011

[64] Shin JH Bog Park S Ho JS. Effects of game-based virtual reality on health-related quality of life in chronic stroke patients: a randomized, controlled study. Comput Biol Med (2015) 63:92–8. doi: 10.1016/j.compbiomed.2015.03.011, PMID: 26046499

[65] Niu Y Sheng S Chen Y Ding J Li H Shi S . The efficacy of group acceptance and commitment therapy for preventing post-stroke depression: a randomized controlled trial. J Stroke Cerebrovasc Dis (2022) 31:106225. doi: 10.1016/j.jstrokecerebrovasdis.2021.106225, PMID: 34837758

[66] Maier M Ballester BR Leiva Bañuelos N Duarte Oller E Verschure P. Adaptive conjunctive cognitive training (ACCT) in virtual reality for chronic stroke patients: a randomized controlled pilot trial. J Neuroeng Rehabil (2020) 17:42. doi: 10.1186/s12984-020-0652-3 32143674 PMC7059385

[67] Levada OA Troyan AS. Poststroke depression biomarkers: a narrative review. Front Neurol (2018) 9:577. doi: 10.3389/fneur.2018.00577 30061860 PMC6055004

[68] Hackett ML Pickles K. Part I: frequency of depression after stroke: an updated systematic review and meta-analysis of observational studies. Int J Stroke (2014) 9:1017–25. doi: 10.1111/ijs.12357, PMID: 25117911

[69] Dayuan Z Lan L Hui C Huanjie L Deliang L Yihui D. The effect of music as an intervention for post-stroke depression: a systematic review and meta-analysis. Complement Ther Med (2022) 71:102901. doi: 10.1016/j.ctim.2022.102901, PMID: 36399968

[70] Epstein FR Liu CM Stevenson JM. Heart transplant recipients prefer a Telemental health cognitive-behavioral therapy intervention delivered by telephone. Telemed J E Health (2019) 25:560–8. doi: 10.1089/tmj.2018.0088, PMID: 30096261

[71] Xianjing Z Jinfeng Z Genying X Meizhen Z Dongwen Y. Effect of Danzhi Xiaoyao powder combined with acupuncture therapy on the expression of BDNF and 5-HT in patients with post-stroke depression. 7/5000. Chin J Gerontol (2019) 39:1562–6. doi: 10.3969/j.issn.1005-9202.2019.07.009

[72] Yücel M Wood SJ Fornito A Riffkin J Velakoulis D Pantelis C. Anterior cingulate dysfunction: implications for psychiatric disorders? J Psychiatry Neurosci (2003) 28:350–4. PMID: 14517578 PMC193981

[73] Murrough JW Abdallah CG Anticevic A Collins KA Geha P Averill LA . Reduced global functional connectivity of the medial prefrontal cortex in major depressive disorder. Hum Brain Mapp (2016) 37:3214–23. doi: 10.1002/hbm.23235, PMID: 27144347 PMC4980239

[74] Li R Ma Z Yu J He Y Li J. Altered local activity and functional connectivity of the anterior cingulate cortex in elderly individuals with subthreshold depression. Psychiatry Res (2014) 222:29–36. doi: 10.1016/j.pscychresns.2014.02.013 24656767

[75] Deng D Liao H Duan G Liu Y He Q Liu H . Modulation of the default mode network in first-episode, drug-naive major depressive disorder via acupuncture at Baihui (GV20) acupoint. Front Hum Neurosci (2016) 10:230. doi: 10.3389/fnhum.2016.00230, PMID: 27242492 PMC4869560

[76] Liu J Fang J Wang Z Rong P Hong Y Fan Y . Transcutaneous vagus nerve stimulation modulates amygdala functional connectivity in patients with depression. J Affect Disord (2016) 205:319–26. doi: 10.1016/j.jad.2016.08.003, PMID: 27559632

[77] Xinhua X Suxin T Sunlin C. Observation on the curative effect of Sini decoction added flavor and Du pulse moxibustion on 30 cases of depression. Clin Res TCM (2021) 13:57–9. doi: 10.3969/j.issn.1674-7860.2021.16.017

[78] Kehua Y Runxue R Wentao F Qian W. Analysis on the compatibility of acupuncture in the treatment of post-stroke depression. J Cardio-Cerebrovascular Diseases Integr Chin Western Med (2023) 21:421–5. doi: 10.12102/j.issn.1672-1349.2023.03.006

[79] Ling C Yan M Shumao L Xuan L Jianbin Z Jinqian L. To explore the clinical study of acupuncture and medicine simultaneously treatment of post-stroke depression (PSD). Chin J Basic Med Chin Med (2019) 25:539–42.

[80] Dongsheng G Yi M Yafang L Yinglin C. Observation on the curative effect of acupuncture and medicine combined with comprehensive program in the treatment of community post-stroke depression. Liaoning J Tradit Chin Med (2017) 44:2575–7.

[81] Chaoda L Chenyang Q Boxuan L Shizhe D Zhihong M. Research progress of time, frequency, direction and depth in the quantitative study of acupuncture manipulation. Clin J Acupuncture (2023) 39:105–10. doi: 10.19917/j.cnki.1005-0779.023041

[82] Tingting Z Falcatus P Yuzhu Q Fang Z. Brief discussion on the sustained effect of acupuncture and its influencing factors. Clin J Acupuncture (2015) 31:1–3.

[83] Xiaofang L Xinhao Z Guotai L. Effects of nurse-led group counseling on psychological distress and self-perceived burden in patients with advanced lung cancer. Oncology Clinical Rehabil China (2019) 26:208–11. doi: 10.13455/j.cnki.cjcor.2019.02.23

[84] DeRubeis RJ Hollon SD Amsterdam JD Shelton RC Young PR Salomon RM . Cognitive therapy vs medications in the treatment of moderate to severe depression. Arch Gen Psychiatry (2005) 62:409–16. doi: 10.1001/archpsyc.62.4.409, PMID: 15809408

[85] Szabadi E. Functional organization of the sympathetic pathways controlling the pupil: light-inhibited and light-stimulated pathways. Front Neurol (2018) 9:1069. doi: 10.3389/fneur.2018.01069 30619035 PMC6305320

[86] Huixin Z Liping Z Yuhong W Hu C Chul YS Huaqiang C . Research progress on ischemic heart disease and autonomic nerve regulation caused by circadian rhythm disturbance. Chin J Senile Cardio-Cerebrovascular Diseases (2021) 23:433–5. doi: 10.3969/j.issn.1009-0126.2021.04.026

[87] Scheer F Chellappa SL Hu K Shea SA. Impact of mental stress, the circadian system and their interaction on human cardiovascular function. Psychoneuroendocrinology (2019) 103:125–9. doi: 10.1016/j.psyneuen.2019.01.016, PMID: 30682628 PMC6686856

[88] Qaseem A Kansagara D Forciea MA Cooke M Denberg TD. Management of Chronic Insomnia Disorder in adults: a clinical practice guideline from the American College of Physicians. Ann Intern Med (2016) 165:125–33. doi: 10.7326/M15-2175, PMID: 27136449

[89] Pan J Wenjie T Tiansheng Z Ye F Jincai H. A survey of personal beliefs about and attitude towards sleep. J Practical Med (2011) 27:118–20. doi: 10.3969/j.issn.1006-5725.2011.01.051

[90] Zhen W. Advances in cognitive behavioral therapy for insomnia. Cardiovasc Disease Electr J Integr Tradit Chin Western Med (2020) 8:20.

[91] Qinghua D Xuejun L Pear K. Research progress of cognitive behavioral therapy for insomniacs. Chinese Convalescent Med (2018) 27:1146–8. doi: 10.13517/j.cnki.ccm.2018.11.009

[92] Xian W Aihua L. Advances in the mechanism of sleep regulation by vagus nerve stimulation. J Stroke Neurol Disorders (2015) 32:1140–1.

[93] Apolinário-Hagen J Drüge M Fritsche L. Cognitive behavioral therapy, mindfulness-based cognitive therapy and acceptance commitment therapy for anxiety disorders: integrating traditional with digital treatment approaches. Adv Exp Med Biol (2020) 1191:291–329. doi: 10.1007/978-981-32-9705-0_17 32002935

[94] Zhao XX Cui M Geng YH Yang YL. A systematic review and meta-analysis of randomized controlled trials of palliative care for pain among Chinese adults with cancer. BMC Palliat Care (2019) 18:69. doi: 10.1186/s12904-019-0456-z 31395039 PMC6688327

[95] Dong W Zhou H Wu R He X Chen X Zhou H . Acupuncture methods for insomnia disorder in the elderly: protocol for a systematic review and network meta-analysis. Syst Rev (2023) 12:124. doi: 10.1186/s13643-023-02287-1 37452408 PMC10347792

