# Supplementary material for: Effect of nonpharmacological interventions on poststroke depression: a network meta-analysis
Source: Front Neurol. 2024 Apr 5;15:1376336. doi: 10.3389/fneur.2024.1376336 (PMC11027129; doi:10.3389/fneur.2024.1376336)
Supplement: Supplementary file 1 [file Data_Sheet_1.zip › Data Sheet 1/Appendix 2-Risk of bias summary.pdf]

	Random sequence generation (selection bias)	Allocation concealment (selection bias)	Blinding of participants and personnel (performance bias)	Blinding of outcome assessment (detection bias)	Incomplete outcome data (attrition bias)	Selective reporting (reporting bias)	Other bias
Chen 2018	+	+	?	?	?	?	?
Cui 2007	+	+	?	?	?	?	?
Du 2017	+	+	−	−	+	+	+
Fang 2020	+	+	−	−	?	?	?
Huang 2012	+	+	+	+	+	+	+
Huang 2018	+	+	−	−	?	?	?
Li 2015	+	?	−	−	?	?	?
Li 2019	+	?	−	−	?	?	?
Li 2020	+	+	−	−	+	+	+
Li 2021	+	+	?	?	?	?	?
Lin 2016	+	+	?	?	?	?	?
Liu 2016	+	+	−	−	?	+	+
Liu 2016.	+	+	−	−	?	?	+
Liu 2021	+	+	?	?	?	?	?
Liu 2021.	+	+	−	−	?	?	?
Liu 2021..	+	+	?	?	?	?	?
Lu 2012	+	+	?	?	+	+	+
Maier 2020	+	+	+	+	+	+	+
Nie 2020	+	+	?	?	?	?	?
Niu 2021	+	+	+	+	+	+	+
Pei 2020	+	?	−	−	−	?	?
Rao 2021	+	+	?	?	?	?	?
Shin 2015	+	?	?	?	+	+	+
Sun 2020	+	+	?	?	+	+	+
Wang 2006	−	−	?	?	?	?	?
Wang 2017	+	+	?	?	?	?	?
Wang 2018	+	+	−	−	?	?	?
Wang 2019	+	+	−	−	+	+	+
Wang 2022	+	+	+	+	+	+	+
Weng 2012	+	+	−	−	?	?	?
Xiao 2011	+	+	−	−	?	?	+
Xu 2015	+	+	−	−	+	+	+
Xue 2020	+	+	−	−	?	?	?
Yang 2016	?	?	−	−	?	?	?
Zhang 2013	+	+	+	+	?	+	+
Zhang 2016	+	+	−	−	+	+	+
Zhang 2017	+	+	−	−	+	+	+
Zhang 2018	+	+	−	−	−	+	+
Zhou 2016	+	+	−	−	+	+	+
Zhu 2010	+	+	−	−	?	?	?
