# Supplementary figures and images for: Effect of nonpharmacological interventions on poststroke depression: a network meta-analysis
Source: Front Neurol. 2024 Apr 5;15:1376336. doi: 10.3389/fneur.2024.1376336 (PMC11027129; doi:10.3389/fneur.2024.1376336)

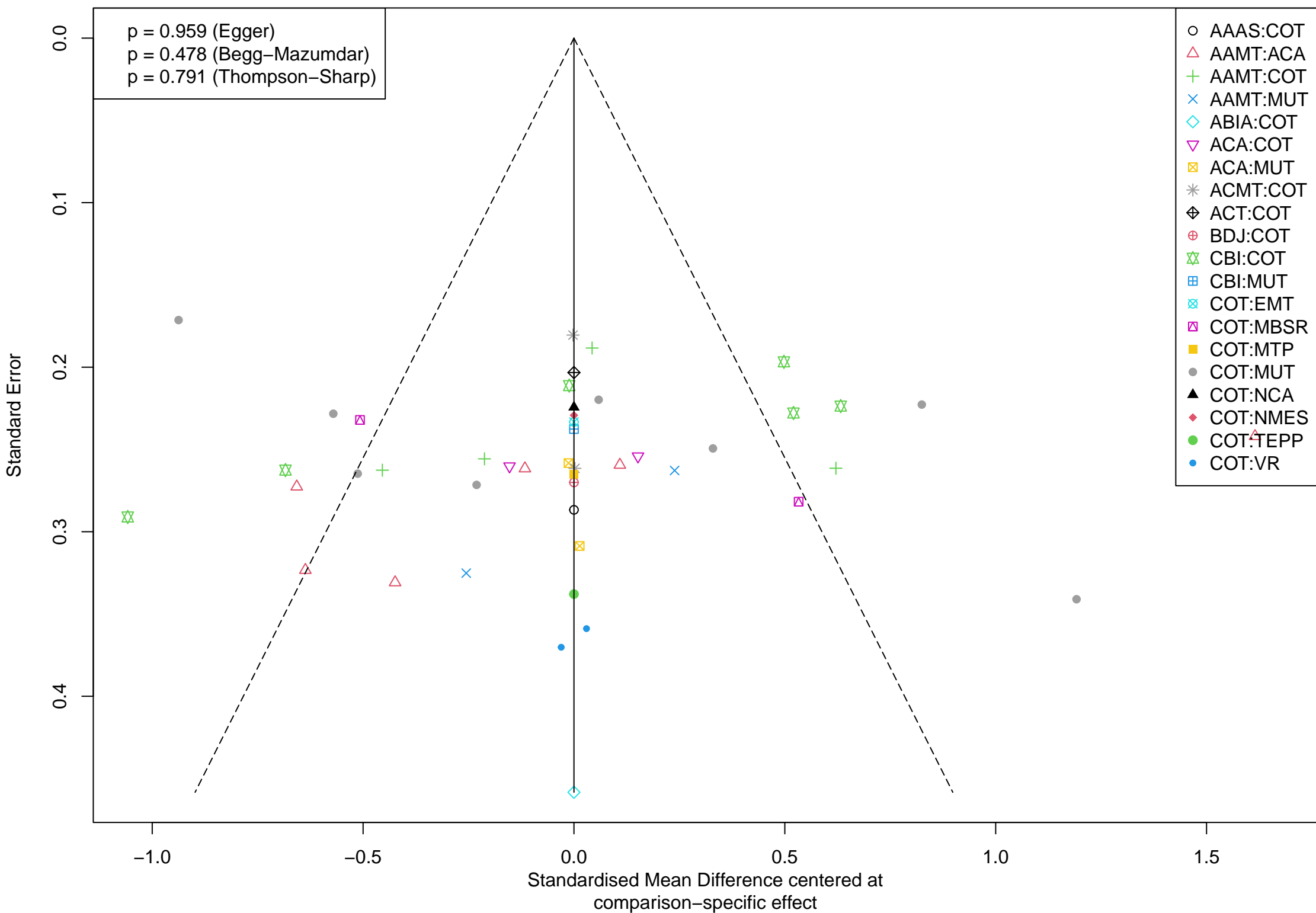

Supplement: Supplementary file 1 [file Data_Sheet_1.zip › Data Sheet 1/Appendix 3.1.Funnel plots.pdf]

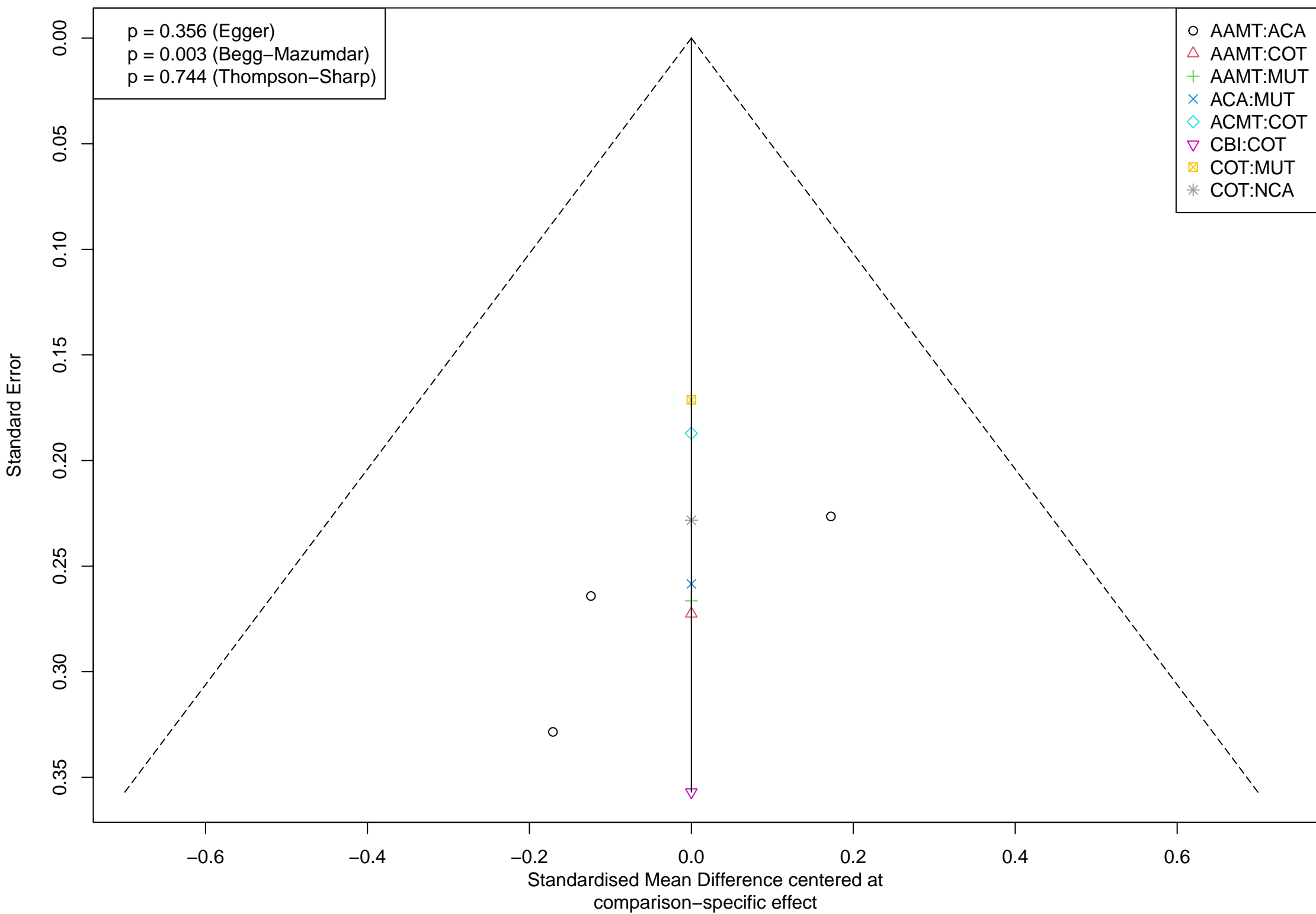

Supplement: Supplementary file 1 [file Data_Sheet_1.zip › Data Sheet 1/Appendix 3.2.Funnel plots.pdf]

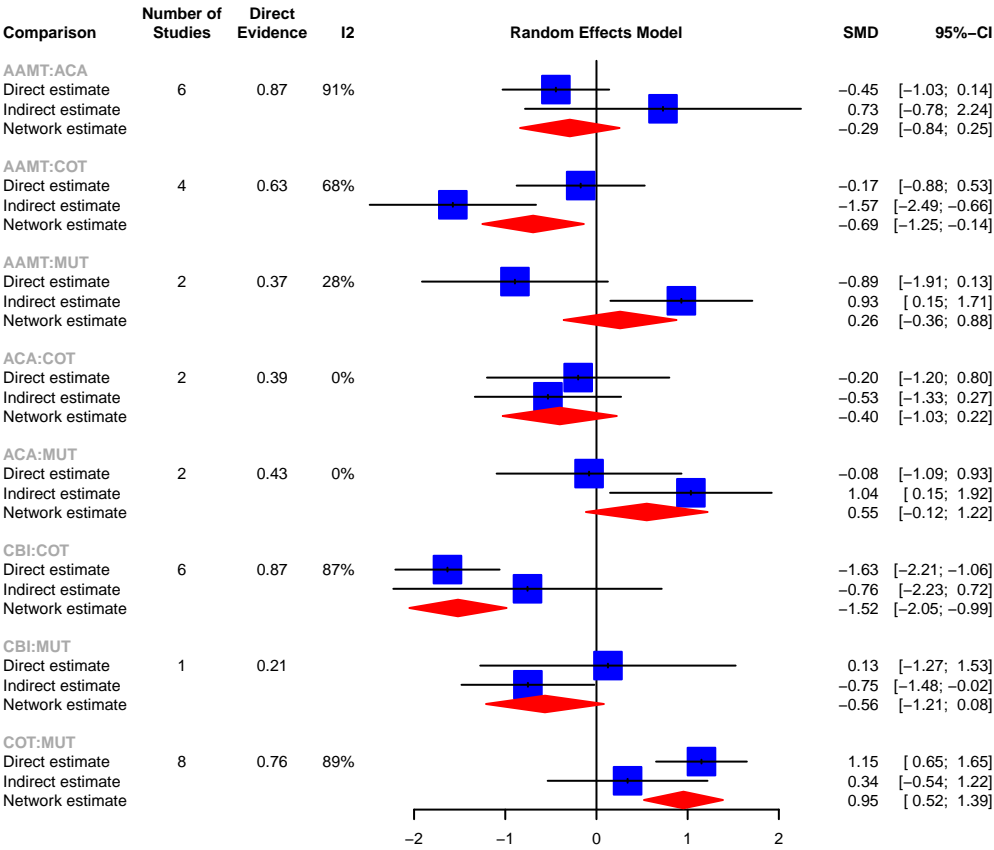

Supplement: Supplementary file 1 [file Data_Sheet_1.zip › Data Sheet 1/Appendix5.1.Direct comparisons.pdf]

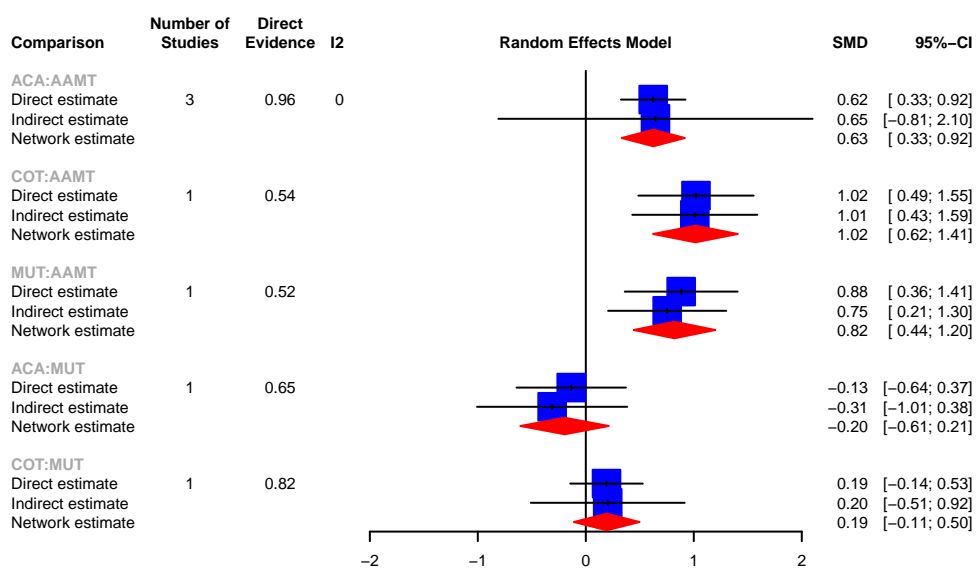

Supplement: Supplementary file 1 [file Data_Sheet_1.zip › Data Sheet 1/Appendix5.2.Direct comparisons.pdf]
